# Longevity and GAPDH Stability in Bivalves and Mammals: A Convenient Marker for Comparative Gerontology and Proteostasis

**DOI:** 10.1371/journal.pone.0143680

**Published:** 2015-11-30

**Authors:** Stephen B. Treaster, Asish R. Chaudhuri, Steven N. Austad

**Affiliations:** 1 Barshop Institute for Longevity and Aging Studies, The University of Texas Health Science Center at San Antonio, San Antonio, Texas, United States of America; 2 Department of Molecular Medicine, The University of Texas Health Science Center at San Antonio, San Antonio, Texas, United States of America; CNRS, FRANCE

## Abstract

**Background:**

Comparative aging studies, particularly those that include species of exceptional resistance to aging processes, can potentially illuminate novel senescence-retarding mechanisms. In recent years, protein homeostasis (proteostasis) has been implicated in fundamental aging processes. Here we further evaluate the relationship between proteostasis and longevity in a selection of bivalve mollusks and mammals with maximum longevities ranging from 3 to 507 years.

**Methods & Results:**

We experimentally examined proteostasis using glyceraldehyde-3-phosphate dehydrogenase (GAPDH) as a reporter, as it is ubiquitously expressed, highly conserved, and conveniently assayed. The ability to maintain this enzymatic function was tested with increasing concentrations of the chaotropic agent urea, revealing a robust relationship with longevity in bivalves and mice. While our shortest-lived mollusk and mouse lost all activity by 2.5 and 3.5 M urea respectively, the longest-lived mollusk species, *Arctica islandica*, still preserved 45% of its basal function even at 6 M urea. To confirm that GAPDH proteostasis has a broad association with longevity, we also investigated a selection of primate species ranging in maximum longevity from 22 to 122 years. They outperformed the mouse at all concentrations, but among the primates results were variable at low urea doses. Still, at 6 M urea baboon and human samples retained 10% of their activity while both mouse and marmoset samples had no activity.

**Mechanism of Exceptional Stress Resistance:**

To explore possible mechanisms of the exceptional stress resistance of *A*. *islandica* GAPDH we enzymatically removed post-translational glycosylation, but observed no decrease in stability. We also removed molecules smaller than 30 kDa, which includes most small heat shock proteins, but again did not compromise the exceptional stress resistance of *Arctica* GAPDH.

**Conclusion:**

While the mechanism underlying *A*. *islandica*’s exceptional stress resistance remains elusive, this research identifies an experimental system that may reveal hitherto unknown mechanisms of protein homeostasis.

## Introduction

Exceptionally long-lived species possess a wealth of untapped research potential for gerontology. Novel aging models are emerging with a variety of useful traits, and when combined with the rapid influx of sequencing information these species are an opportunity to elucidate successful aging strategies. Traditional animal models are short-lived and highly susceptible to basic aging processes. They deteriorate and die rapidly which from one perspective enhances their experimental utility. However, humans are already highly resistant to basic aging processes, so the senescence-retarding interventions effective in traditional models may not be as successful at improving and prolonging human health. It is in exceptionally long-lived species that we may discover mechanisms of exceptional senescence-resistance.

*Arctica islandica*, the ocean quahog, is one such species. With a maximum lifespan of over five hundred years [1,2] it may contain information relevant for combating senescence and age-related diseases. These lifespans can be authenticated in natural populations via schlerochronology–the counting of annual growth rings in the shell [3,4]–a significant advantage of bivalves over many other model organisms. By analyzing these growth rings, a remarkable range of longevities have been determined. The bivalve species utilized in this study have maximum lifespans ranging from 7 to 507 years, yet are all bottom dwelling, filter feeding heterodont bivalves of comparable size. With many ecological similarities it is easier to identify salient differences between these species that may contribute to their variable aging rates. Combined with their low-cost commercial availability and ease of care, bivalves are emerging as an ideal experimental system for comparative analysis of aging processes [5–8].

The progressive loss of protein homeostasis, now called proteostasis, has arisen as a likely contributor to the progressively deteriorating aging phenotype. Proteostasis is the maintenance of a functional proteome through strict regulation of translation, folding, modification, trafficking and degradation. Each of these processes are measurably compromised with age [9–11], leading to cytotoxic aggregates [12], impaired cellular function and contribute to the variety of pathologies we experience with age [13–18]. Notably, neurodegenerative diseases such as Alzheimer's and Parkinson's are characterized by the insoluble accumulation of proteins and are undeniably age-related [19]. Experimental longevity manipulations have further implicated the collapse of proteostasis in senescence processes. Interventions that extend lifespan, such as rapamycin, caloric restriction, and insulin-like signaling pathway manipulation each have a pronounced reduction in protein aggregation [20–23] and oxidative damage markers [24,25] along with increased autophagy [26,27] and molecular chaperone activity [28,29]. Longevity appears to be highly correlated with the effectiveness of the proteostasis network. Indeed, the common *daf-2* mutation that extends lifespan in *C*.*elegans* absolutely requires HSF1 [30], a master regulator of molecular chaperone expression, key players in proteostasis [31].

We have previously shown that among species of bivalve mollusks, longer-lived species are more resistant to global proteome unfolding and better maintain creatine kinase activity when confronted with misfolding stressors. We also showed that the proteome was dramatically less likely to form large aggregates under heat stress in long-lived species such as *A*. *islandica*, compared with shorter-lived species, including the short-lived mouse. We also found that a reporter protein, FITC-tagged bovine serum albumin, was less prone to heat-induced aggregation in tissue lysates of long-lived bivalve species in comparison with shorter-lived bivalves. [5]

In order to extend our investigations of the relation between species longevity and relative proteostatic capability, we exploited glyceraldehyde 3-phosphate dehydrogenase (GAPDH) as a representative protein. GAPDH is an abundant protein commonly known for catalyzing the sixth step of glycolysis, but has been implicated in a range of other cellular functions. It is constitutively expressed, highly conserved, and absolutely essential. It is mostly known for its classical housekeeping role in glycolysis, for which it can be easily assayed. When provided with its substrates glyceraldehyde 3-phosphate and NAD^+^, GAPDH will catalyze the reaction to D-glycerate 1,3-bisphosphate and NADH. This reaction can be conveniently monitored with a spectrophotometer in real time. However, if the protein has been damaged or unfolded by stressors, this function will be inhibited, reducing the rate of production. We utilized this representative enzymatic activity as an accessible marker of cellular proteostasis to determine each species' relative ability to maintain protein structure and function when stressed. Resisting the deleterious effects of age likely requires the ability to maintain an intact and highly functional proteome, while enduring the variety of internal and external stressors assured with time [9,32].

We assessed GAPDH activity under urea misfolding stress in four species of bivalves ranging in maximum longevity from 7 to 507 years along with the laboratory mouse as a short-lived mammalian standard. In addition, to determine whether the same relation between proteostatic capacity and longevity exists among species more closely related to humans, we performed the same analysis in three species of primates ranging in maximum longevity from 22 to 122 years, again alongside the mouse. We also assessed whether small metabolites, small heat shock proteins, or post-translational glycosylation might play a role in stabilizing GAPDH under stress.

## Materials and Methods

### Species Included

Four species of bivalve mollusks were included: *Ruditapes phillipanarum* (maximum reported longevity, L_max_, = 7 years [33]), *Callista chione* (L_max_ *=* 30 years [34]*)*, *Mercenaria mercenaria* (L_max_ *=* 106 years [35]) and *Arctica islandica* (L_max_ = 507 years[1,2]). Individuals were collected opportunistically from commercial fishermen working the offshore waters of the state of Washington in the United States (*Ruditapes*), Croatia (*Callista*), and the state of Massachusetts (US) and Wales (*Mercenaria* and *Arctica*). These species were chosen for their disparate longevities of course, but also for their ecological similarities. They employ similar lifestyles, filter-feeding microorganisms with an extended siphon while their bulk is burrowed beneath the ocean sediment, additionally protected by their thick valves. They are of comparable size, ranging from a maximum shell height of 60 mm (*Ruditapes*) to 120 mm (*Mercenaria*) and enjoy overlapping climate preferences of 12-24°C (*Ruditapes*) to 5–15°C (*Arctica*). No permissions were required to collective bivalves and their tissues. It should be noted that we are testing for phylogenetic independent effects, as there is no obvious phylogenetic relation to longevity; the shortest-lived and second to longest-lived are the closest relatives[5]. In order to bring our results a medically relevant perspective we also tested a trio of primates with disparate longevities: common marmoset (*Callithrix jacchus*, L_max_ = 22 years [36]) baboon (*Papio hamadryas*, *L*_*max*_ = 38 years [37]) and human (*Homo sapiens*, L_max_ = 122 years). Additionally, we included the common aging model C57BL/6 mouse (*Mus musculus*, L_max_ = 3 years). We are thus investigating our hypothesis in both bivalves and mammals, but the chance to compare the most successful aging model against the most common aging model should not be understated. While such a comparison across phyla is of questionable relevance, placing each group on the same proteostasis scale could be enlightening.

### Ethics Statement

All procedures and protocols used in this entire study were approved by their respective animal or human use committee. In addition, all mammalian tissues were obtained from samples taken for purposes unrelated to this study. Specifically, human muscle tissue was obtained from volunteers in a diabetes study (samples were from controls) with full written informed consent, approved by the University of Texas Health Science Center San Antonio Institutional Review Board. Mouse and marmoset muscle tissue were obtained from captive colonies used in aging research at the University of Texas Health Science Center San Antonio and the Texas Biomedical Research Institute, respectively. Baboon tissue was obtained from the captive colonies at the Southwest National Primate Research Center, within the Texas Biomedical Research Institute. Euthanasia methods were those approved by the American Veterinary Medical Association for these species. Each protocol was approved either by the University of Texas Health Science Center San Antonio Institutional Animal Care and Use Committee (mice), and the use of baboon tissue was approved by the Texas Biomedical Research Institute Institutional Animal Care and Use Committee.

### Tissues Used

For the bivalves, adductor muscle samples were obtained from wild caught individuals provided by collaborators at the Virginia Institute of Marine Science and the School of Ocean Sciences in Bangor, Wales. The adductor muscle holds the valves closed, maintaining the bivalve's only defense against the ocean's host of predators. This function must be preserved for successful aging as a prey animal. Individual ages were determined by our collaborators via counting annual growth rings in the shell [3,4] and young adults were chosen from each species. Young adults should have minimal age-related degeneration yet prevent any developmental stage dependent results between species; the longer-lived species have not reached sexual maturity by the time the shorter-lived species have died. Thus we used individuals of approximately 3, 10, 20 and 35 years of age for *Ruditapes*, *Callista*, *Mercenaria* and *Arctica* respectively, based on availability and maturation age. They were of unknown sex. Unfortunately, young adult samples for the three primates were not available and older individuals had to be incorporated into the study. The marmoset and baboon samples were approximately 7 and 17 years of age respectively and of mixed sex. The human samples were of unknown age and unknown sex. For the mammals, skeletal muscle was chosen as the functionally equivalent tissue to the bivalve adductor. Age related decreases in mammalian muscle function are well-documented [38] and, like the bivalves, impairing this tissue would be an obvious detriment to survival and longevity in the wild. In order to delay these consequences, exceptionally long-lived species likely utilize superior proteostasis to prevent or ameliorate these age-related declines in function and its consequent increase in mortality.

### GAPDH Activity

Common enzymatic activities can be employed as convenient representatives of overall proteome homeostasis. Many essential enzymes have well defined substrates, active sites and overall functions which are easily probed. These catalysts are completely dependent on their complex three dimensional structure, which if disrupted, will cause a commensurate decrease in activity. We utilized urea as chaotropic agent, disrupting hydrogen bonding and weakening the hydration shell critical for protein folding [39]. At low concentrations, active sites and overall conformation will be mildly disrupted. High concentrations can denature the structure completely. We probed each species' ability to resist these disruptions and maintain enzymatic activity as a marker for their relative proteostasis. GAPDH function was measured and represented as the fold change in activity from basal levels to stressed.

In brief, adductor muscle (bivalves) or skeletal muscle (mammals) was homogenized in 15mM sodium pyrophosphate buffer containing 30mM sodium arsenate at pH 8.5, with a protease inhibitor cocktail to maintain sample integrity after homogenization. Ultracentrifugation at 100,000g for one hour was used to isolate the cytosolic fraction. From this, equal amounts of protein, as determined by BCA, were loaded into reaction mixtures containing the following: 0.3mM NAD^+^, 0.3mM D-glyceraldehyde-3-phosphate, 4mM DTT, and 3.5ug/uL lysate. Cuvettes were read in a spectrophotometer and the _Δ_A340/minute corresponding with the GAPDH dependent reduction of NAD^+^ to NADH is determined from the initial linear response. This is a conventional GAPDH enzyme activity assay [40,41]. The conditions described yielded measurable baseline activity to which stressed levels could be compared. When stressors were included, they were added twenty minutes prior to the addition of D-glyceraldehyde-3-phosphate, effectively pre-stressing the enzyme in that species' lysate before the activity was assayed.

To narrow and identify potential facilitators of GAPDH stability the samples were rinsed through a centricon filter six times, isolating the representative enzyme from anything less than 30kDa. This small molecule depleted lysate was then utilized as previously described, but with the small molecules lacking, their influence on GAPDH stability could be analyzed. The effects of glycosylation post-translational modification (PTMs) were also of interest, as these are well known to enhance protein stability [42,43]. Samples were incubated in a digestive enzyme cocktail (Sigma-Aldrich: EDEGLY-KT) designed to remove all N-linked and simple O-linked carbohydrates from the sample prior to the stressed GAPDH activity assay.

### Statistical Analysis

Statistics were performed using JMP 9.0 software (SAS, Cary, NC, USA). Data are visualized as means, error bars are +/- 1 SEM. Differences among species were assessed by ANOVA. Comparisons notated with asterisks are statistically different by *post hoc* testing using Tukey’s HSD, p < 0.05 (*), 0.001 (**), and 0.0001(***). Raw data for all figures found in S1 Table.

## Results

### GAPDH Activity

**Fig 1:**
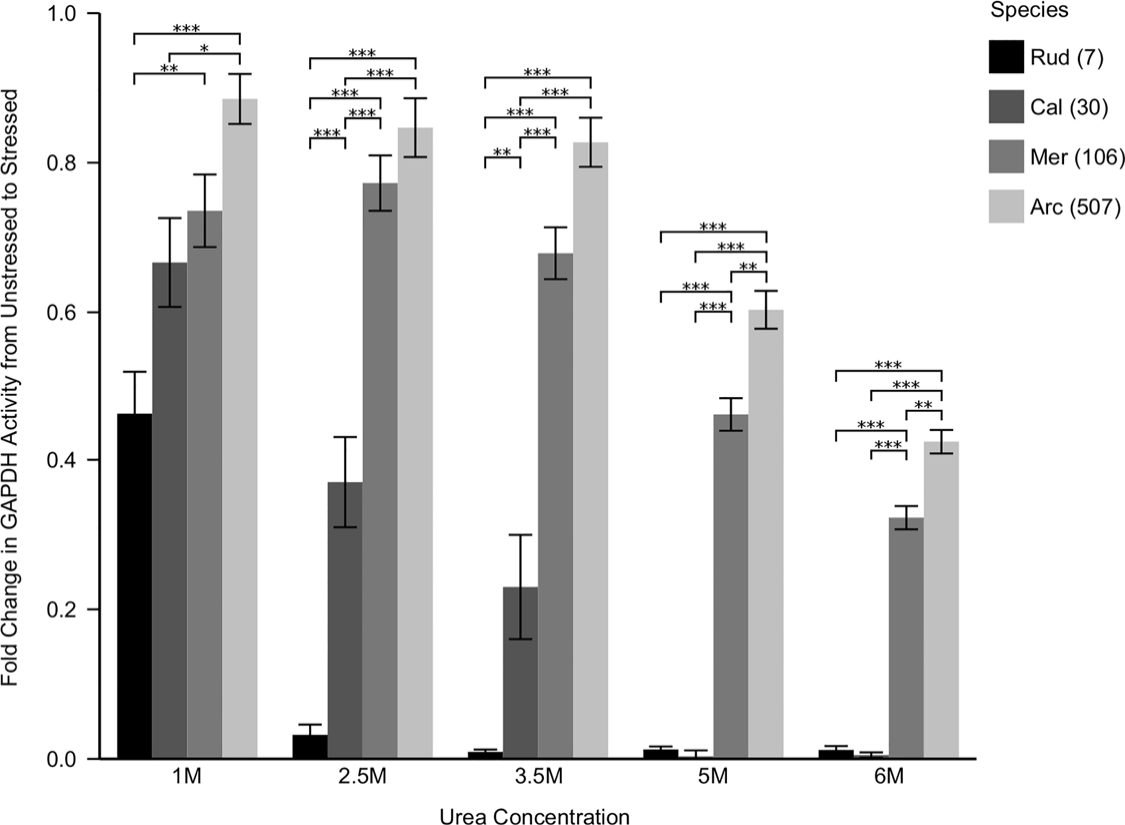
GAPDH Activity with Increasing Urea Stress in Bivalves. Lysates from each species were pre-stressed for twenty minutes in the noted urea concentrations. Glyceraldehyde 3-phosphate was then added, and the activity of endogenous GAPDH was monitored as ΔA_340_/minute, corresponding to the reduction of NAD^+^ to NADH. Data is reported as the fold change from unstressed activity. Differences among species were assessed by two way analysis of variance indicating a significant main effect of species (F_3,60_ = 23.7, p < 0.0001) stress (F_4,60_ = 30.9, p < 0.0001), as well as the interaction between species and stress (F_12,60_ = 10.2, p < 0.0001). Long-lived species maintain GAPDH function at all doses tested, while shorter-lived species were dramatically compromised. Asterisks indicate significant individual differences as assessed *post hoc* with Tukey's HSD, p < 0.05 (*), 0.001 (**), and 0.0001 (***), respectively. Rud = *Ruditapes*, Cal = *Callista*, Mer = *Mercenaria*, Arc = *Arctica*. Numbers in parentheses are maximum species longevity in years.

**Fig 2:**
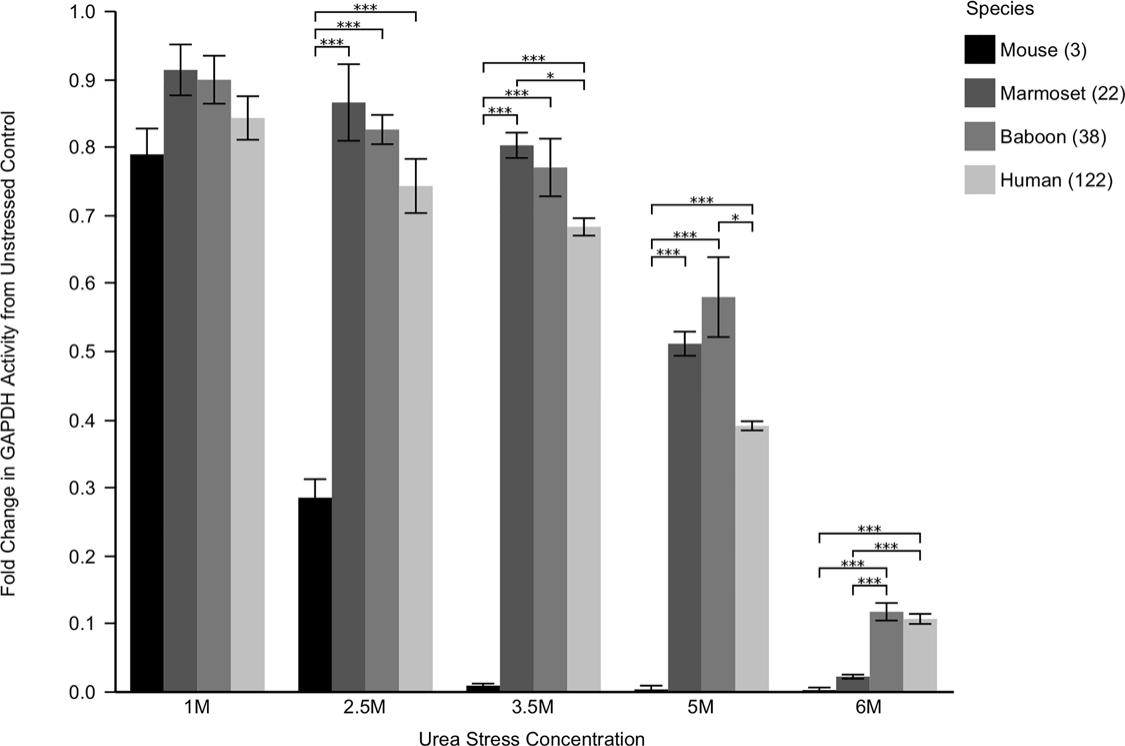
GAPDH Activity with Increasing Urea Stress in Mammals. Lysates from each species were pre-stressed for twenty minutes in the noted urea concentrations. Glyceraldehyde 3-phosphate was then added, and the activity of endogenous GAPDH was monitored as ΔA_340_/minute, corresponding to the reduction of NAD^+^ to NADH. Data is reported as the fold change from unstressed activity. Differences among species were assessed by two way analysis of variance indicating a significant main effect of species (F_3,60_ = 222.5, p < 0.0001) stress (F_4,60_ = 429.9, p < 0.0001), as well as the interaction between species and stress (F_12,60_ = 26.1, p < 0.0001). Long-lived species maintain GAPDH function at all doses tested, while shorter-lived species were dramatically compromised. Asterisks indicate significant individual differences as assessed *post hoc* with Tukey's HSD, p < 0.05 (*), 0.001 (**), and 0.0001(***), respectively. Numbers in parenthesis are maximum species longevity in years.

In order to assess each species' relative proteostasis potential, GAPDH activity was monitored under increasingly stressed conditions and quantified as the fold change from basal levels. As a representative of global proteome stability, protection of GAPDH activity was robustly correlated with longevity in both bivalves and mammals. While GAPDH in short-lived *Ruditapes* lost all activity by 2.5 M urea, and mouse showed negligible activity by 3.5 M urea, the exceptionally long-lived *Arctica* maintained 45% of its basal activity in 6 M urea (Fig 1). Long-lived mammals also performed well, with human and baboon samples maintaining 10% of their basal activity at 6 M urea (Fig 2), significantly outperforming the other, shorter-lived mammals. However, at lower doses, the shorter-lived primate's GAPDH was more stable than the human samples. At all doses the commonly used but short-lived C57Bl6 was the least stable mammal, and lost all activity by 3.5 M urea. It should be noted that the long-lived bivalves retained greater GAPDH activity relative to unstressed controls than even the human sample. This suggests that long-lived bivalves may possess more effective proteostasis mechanisms than even the longest-lived mammals.

### Small Molecule and Glycosylation Removal

**Fig 3:**
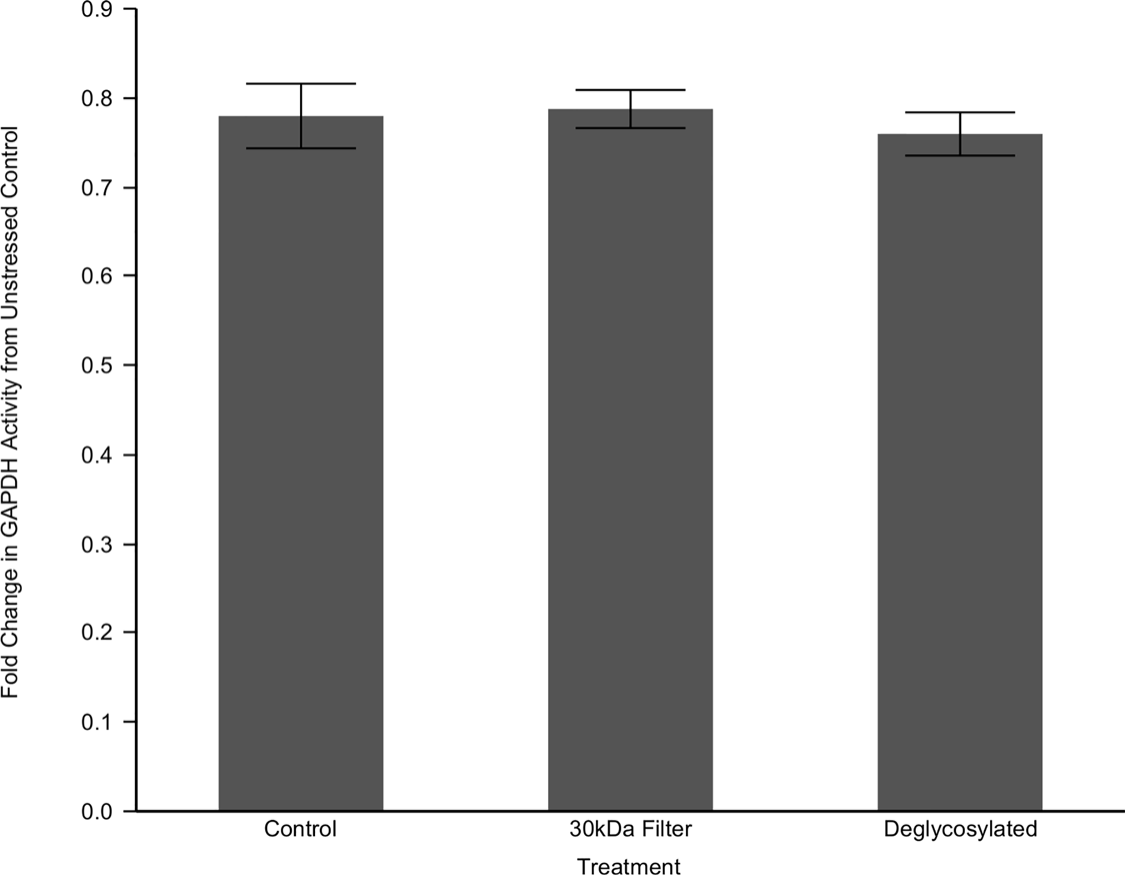
Small Molecule and Glycosylation Effects on GAPDH Stability in *Arctica islandica*. Lysates from *Arctica islandica* were filtered through a 30kDa centricon, isolating GAPDH from any potential small stabilizers. Another sample was treated with a deglycosylation kit to remove N-linked and O-linked carbohydrate modifications. These depleted lysates were pre-stressed for twenty minutes in 3.5 M urea. Glyceraldehyde 3-phosphate was then added, and the activity of endogenous GAPDH was monitored as ΔA_340_/minute, corresponding to the reduction of NAD^+^ to NADH. Data is reported as the fold change from unstressed activity. GAPDH stability was not further compromised by deglycosylation (p = 0.62) or the removal of small molecules (p = 0.85) as compared to the unmodified control.

The remarkable protein stability demonstrated by muscle lysate of *Arctica* raises the obvious question of what stabilizing components they employ that shorter-lived species lack. Two possibilities are small heat shock proteins and/or small metabolites may contribute to this stability. In order to examine these possibilities, we isolated them from the system by running the lysate through a 30kDa centricon, removing cellular components less than 30kDa in size. This should remove stabilizing metabolites and monomers of small heat shock proteins. This depleted fraction was utilized for the GAPDH assay as before, comparing 3.5 M urea stressed activity to basal levels. We focused our attention on *A*. *islandica* as it exhibited the most robust result in response to the urea. If small proteins or other molecules are major contributors to this result, their removal should increase GAPDH's susceptibility to urea stress. Surprisingly, there was no significant difference in *Arctica's* GAPDH stability under this urea stress when depleted of components less than 30kDa in size (Fig 3).

Considering the lack of an effect from small molecules, we next hypothesized that long-lived species may employ certain post-translational modifications to stabilize protein structure and maintain function. Glycosylation modifications have a variety of structural consequences depending on their type and location, but can include improved tertiary stability. We applied a commercial deglycosylation kit to globally remove all N-linked and O-linked carbohydrate post-translational modifications. This enzyme suite was applied to the lysate at manufacturer specifications and the experiment re-run with 3.5 M urea stress as before. Again, there was no significant difference in *Arctica's* GAPDH stability when compared to the unmodified lysate (Fig 3).

## Discussion

We have shown that the exceptionally long-lived bivalve mollusk species, *Arctica islandica*, is exceptionally successful at preserving its GAPDH activity in muscle compared with shorter-lived species of bivalve mollusks, mice and primates. Combined with our previous work demonstrating that its proteome is less subject to misfolding, aggregation and also that it preserves the activity of creatine kinase as compared with shorter-lived species [5], we can conclude that *Arctica* has evolved exceptionally effective mechanisms for protecting its proteome from stress. If we can identify the mechanism(s) by underlying this stability, it could have dramatic implications for interventions in human protein folding diseases and also potentially for aging itself.

One obvious mechanism of superior proteome protection would be a superior system of molecular chaperones, which are key components of the proteostasis network [31]. A robust and adaptive suite of chaperones present in lysates from long-lived species' muscle could bind and stabilize GAPDH to maintain functional conformation in the presence of misfolding stress. However, the large chaperone families that would be expected to mediate this effect–heat shock protein 60 (HSP60), HSP70, and HSP90 –are ATP-dependent [44]. ATP was not included in our reaction mixtures, making this scenario unlikely unless long-lived bivalves possess unique large chaperones not dependent on ATP. Provocatively, the one bivalve species with a high quality whole genome sequence–the Pacific oyster (*Crassostrea gigas*)–has 88 HSP70’s compared with ~17 in humans [45]. However, as no large HSPs that do not require ATP are known from other organisms, this possibility should be considered highly remote. Additionally, the possibility of any adaptive stress responses, including all forms of protein turnover such as enhanced autophagy, proteasome activity or accelerated *de novo* translation, should have been eliminated by our homogenization and centrifugation procedures. The 100,000g soluble fraction used in our study represents a “snapshot” of the proteome and its stability without adaptive elements. Within these confines, our previous results indicated this stability is not unique to a single protein. Our long-lived species' entire proteome demonstrated resistance to urea induced unfolding and temperature induced aggregation [5]. The protective mechanism must preserve the proteome indiscriminately.

Our experimental system eliminates many of the most obvious candidates for proteome stabilization but a few remain. Small heat shock proteins (sHSPs) are molecular chaperones [46] that function independent of ATP [47] yet can bind and stabilize misfolded proteins. Indeed, HSP18.1 in peas was found to dodecomerize and bind to GAPDH under heat stress, preventing its aggregation [48]. Additionally, a variety of osmolytes are known to stabilize protein structure [49–51] counteracting hydrostatic pressure, increasing thermostability and even combating denaturization by urea [52]. We evaluated the possibility that similar sHSPsor osmolytes may have been responsible for *Arctica's* high GAPDH stability by running our samples through a 30kDa centricon filter to remove any such small stabilizing molecules. This removal did not affect GAPDH stability, suggesting something larger than 30kDa is the key stabilizer. While many potentially stabilizing metabolites were removed, this data should not be regarded as conclusive evidence of sHSP non-involvement; they may oligomerize beyond the 30kDa filter threshold or may be bound strongly enough to prevent their removal by filtration. We also tested the possible role of glycosylation post-translational modifications, as they have a demonstrated influence on protein stability [42, 43] and GAPDH has a known modification site at Th227 [53]. As there may be novel sites, we removed all N-linked and O-linked glycosylations enzymatically, but did not yield a reduction of GAPDH activity. Other modifications were not probed and could play an important role.

GAPDH stability may be interesting in its own right and not just as a representative for the rest of the proteome. While the phylogenetic distance between bivalves and mammals is great, GAPDH is exceedingly well conserved with ~75% perfect identities between human and invertebrate GAPDH, likely due to its essential role in glycolysis. Beyond this classic housekeeping role, GAPDH has been implicated in a variety of other cellular processes, including cytoskeletal organization, organelle biogenesis and autophagy. Importantly, single nucleotide polymorphisms in the GAPDH gene have been associated with late onset Alzheimer's disease [54]. GAPDH is involved in neuronal apoptosis [55,56] and known to bind both amyloid beta [57] and its precursor protein [58]. As Alzheimer's disease is characterized by the collapse of proteostasis and GAPDH is involved, discovering novel stabilizing components could provide therapeutic targets for further research. These targets could be affirmed in conventional mouse models. Their poor protein stability is an opportunity for intervention and enhancement by translating the superior proteostasis of long-lived models like *Arctica islandica*. Combined with our previous work and other comparative studies, long-lived species' general resilience against protein aggregation is a salient factor of successful aging and is notably lacking in more common models.

While the relationship between proteostasis and longevity was robust in the bivalves, it should be noted that the trio of primates do not fit our hypothesis at all doses. The mammals are firmly rooted by the poor C57BL6 performance, but the marmoset, baboon and human results are inconsistent at lower urea concentrations. At 3.5 M urea, GAPDH activity from human muscle is actually significantly lower than marmoset (p < 0.05) and at 5 M urea baboon GAPDH has greater activity than the other mammals. It is only at 6 M urea, that human and baboon are significantly superior to marmoset. These results are puzzling. We should note that unlike our bivalve samples, our primate samples were acquired from any source (and any muscle) we could find. Consequently, the samples were taken from different muscle groups (mouse and marmoset: gastrocnemius, baboon: masseter, human: vastus lateralis). These different muscle groups are composed of different mixtures of fiber types. Also, unlike our bivalve samples, we were unable to control the relative ages of the individuals from which the samples were obtained. Together, these factors could confound direct comparisons among the primate species. The hypothesized trend of greater GAPDH activity in response to misfolding stress only appears among primates at the highest dose tested.

In sum, we have shown that stress resistance of the proteome is reliably associated with longevity in a selection of bivalve mollusk species ranging over nearly two orders of magnitude. As protein homeostasis is emerging as a key player in the aging process, the success in maintaining proteostasis shown by our longest-lived species, *A*. *islandica*, suggests that it may have evolved proteome stabilizing mechanisms of relevance to developing interventions that slow the aging process in humans.

## Supporting Information

S1 Table Raw data for Figs 1–3. Species are Ruditapes = *Ruditapes phillipanarum*, Callista = *Callista chione*, Mercenaria = *Mercenaria mercenaria*, Arctica = *Arctica islandica*.
(XLSX)
